# Risk Factors for the Recurrence of CVD Incidents in Post-Stroke Patients over a 5-Year Follow-Up Period Based on the ICF Classification

**DOI:** 10.3390/ijerph18116021

**Published:** 2021-06-03

**Authors:** Mateusz Lucki, Ewa Chlebuś, Agnieszka Wareńczak, Przemysław Lisiński

**Affiliations:** 1 Department of Cardiology, Hospital Center of the Jelenia Góra Valley, Poland Ogińskiego Str., No. 6, 58-506 Jelenia Góra, Poland; 2 Department of Rehabilitation and Physiotherapy, University of Medical Sciences, 28 Czerwca 1956 Str., No. 135/147, 60-545 Poznań, Poland; ewachlebus@ump.edu.pl (E.C.); agnieszka.warenczak@ump.edu.pl (A.W.); plisinski@vp.pl (P.L.)

**Keywords:** stroke, CVD, secondary prevention, ICF

## Abstract

*Background and Objectives:* Stroke is a strong risk factor for recurrent cardiovascular disease (CVD) incidents. The risk of post-stroke CVD incidents can be reduced by eliminating the most relevant risk factors. The aim of the study was to compare the incidence of recurrent CVD events and to determine the quantitative and qualitative differences in CVD risk factors over the 5-year follow-up period in patients with ischemic stroke (IS) and haemorrhagic stroke (ICH) with the use of ICF classification categories to present these differences. *Materials and Methods:* The study was retrospective. The study groups included 55 post-IS patients and 47 post-ICH patients. The results were translated into the categories from the International Classification of Functioning, Disability and Health (ICF) classification. *Results:* As compared to post-ICH patients, post-IS patients were significantly more frequently observed to have recurrent CVD incidents (*p* < 0.001), including fatal CVD incidents (*p* = 0.003). More risk factors in total were identified in both post-IS patients (*p* = 0.031) and post-ICH patients (*p* = 0.002) who had a recurrent CVD incident. Post-IS patients were more often found to have arterial blood pressure higher than 140/90 mmHg (*p* = 0.045). On the other hand, post-ICH patients were more frequently observed to have carotid artery stenosis in the range of 50–69% (*p* = 0.028) and an eGFR of <15 mL/min/1.73 m^2^ (*p* = 0.001). *Conclusions:* The type of primary stroke determines the type and incidence of risk factors as well as the recurrence rate of CVD incidents over a 5-year follow-up period. Patients after IS have a higher risk of recurrence of CVD events, including fatal ones in the 5-year follow-up compared to patients after ICH. In addition, post-IS patients who have a recurrent CVD event over a 5-year follow-up have more risk factors for a CVD event than ICH. The ICF classification can be useful for assessing and analysing risk factors for recurrent CVD incidents, which can help to improve the effectiveness of secondary prevention.

## 1. Introduction

Stroke is a serious social problem because it is the second leading cause of death in the world and the leading cause of long-term disability [1]. The risk of death in patients with recurrent stroke is approximately 50% [2]. Mortality due to recurrent stroke is twice as high as in the first stroke event [3]. Moreover, post-stroke patients are at high risk of recurrence of cardiovascular diseases (CVD) [4]. The cumulative risk of a recurrent CVD event ranges from 10% to 12% in the first year and from 30% to 40% over a five-year period. Recurrent stroke, which most often has the same etiopathogenesis as the primary stroke, accounts for more than 75% of the secondary sequelae of CVD. Interestingly, more than 40% of post-haemorrhagic stroke (ICH) patients have ischemic stroke (IS) and only 5% of post-IS patients have ICH as a recurrent CVD incident [2].

Based on analyses carried out by experts of the World Health Organization (WHO), it can be concluded that about 80% of recurrent CVD incidents could be avoided if the most relevant risk factors were eliminated [5]. The generally recognised modifiable risk factors for CVD related to secondary prevention include arterial hypertension, atrial fibrillation, diabetes, dyslipidaemia, abnormal body mass index (BMI), carotid artery disease, depression, insomnia, smoking, and alcohol abuse [6,7,8,9,10,11,12,13,14,15,16,17,18,19,20]. The coexistence of the above risk factors increases the likelihood of a recurrent CVD incident [21]; therefore, it is reasonable to monitor patients for all these risk factors simultaneously.

The International Classification of Functioning, Disability and Health (ICF) makes it possible to compare and identify differences in the assessment of impairments which affect functions and body structures as well as the activity and participation. This assessment is particularly important in the management of post-stroke patients due to the complexity of their psychosomatic dysfunctions. The ICF classification has been approved by the WHO as an international standard for describing health status and health-related conditions [22]. This makes it possible to organise the clinical data contained in medical records and to present them in a clear graphical form. Translating clinical data into the categories from the ICF classification increases the reliability of a complex assessment of a patient’s health status [23]. The usual clinical practice is to use the ICF classification mostly for post-stroke patients at the time of initial diagnosis and during the acute treatment period [24]. The available medical literature contains only a few publications relating to the use of the ICF classification for assessing post-stroke patients in the course of secondary prevention [25].

## 2. Objectives

1. To compare the incidence of recurrent CVD incidents over a 5-year follow-up period in post-IS and post-ICH patients;
2. To determine quantitative and qualitative differences relating to risk factors for CVD in post-IS and post-ICH patients according to the recurrence of CVD incidents over a 5-year follow-up period;
3. To use the categories of the ICF classification as a tool for presenting differences in the incidence of risk factors for recurrent CVD incidents according to previous IS or ICH and the recurrence of CVD incidents over a 5-year follow-up period.

## 3. Materials and Methods

### 3.1. Study Design/Study Groups/Inclusion and Exclusion Criteria

**Figure 1:**
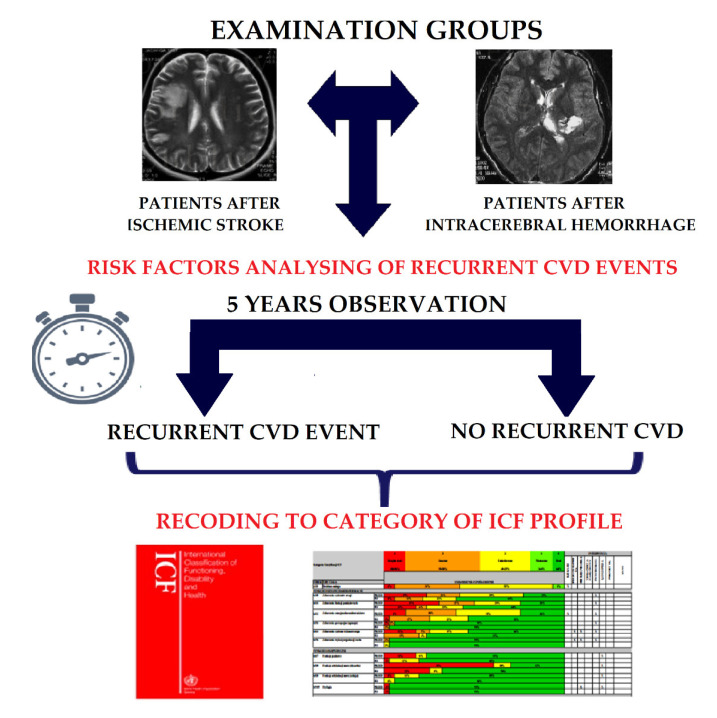
Study design. CVD—cardiovascular disease; ICF—International Classification of Functioning, Disability and Health.

The study was retrospective and was conducted in the Neurological Rehabilitation Unit of the Rehabilitation Department at Wiktor Dega Orthopedic and Rehabilitation Teaching Hospital attached to K. Marcinkowski Poznań University of Medical Science in the period from 1 January 2015 to 31 December 2016. In this period in the Neurological Rehabilitation Unit were hospitalized 258 patients after stroke, including 169 after IS and 89 after ICH. The study was divided into 2 stages. The first stage was to analyse the frequency of risk factors for a recurrent CVD event based on the patient’s medical history. After the inclusion and exclusion criteria were applied, 189 patients were enrolled in this stage, including 109 after IS and 80 after ICH. The second stage was to analysed the frequency of recurrent CVD events within five years of the first stroke episode based on contact with the patient or a close family member authorized to provide information about the patient’s health status. Finally, 55 patients after IS and 47 patients after ICH were qualified for the second stage. The results were translated into categories of the ICF classification and are presented in graphical form. A detailed study diagram is shown in Figure 1.

The study was approved by the Ethics Committee attached to K. Marcinkowski Poznań University of Medical Sciences (Approval No. 174/21 of 11 March 2021). The study was conducted in accordance with the ethical principles for biomedical research as stated in the Declaration of Helsinki. The study was registered in the Clinical Trial Registry: NCT04590287 https://clinicaltrials.gov/ct2/show/NCT04590287 (accessed on 19 October 2020).

The study inclusion criteria were as follows: (1) patients with stroke as confirmed by medical imaging, (2) patients hospitalised within 14 days of the stroke in the neurological rehabilitation department, (3) full medical records containing detailed information on all possible risk factors, (4) current medical records to confirm whether there was a recurrent CVD incident over the 5-year follow-up period, and (5) use of the prescribed pharmacotherapy of chronic diseases by patients.

The study exclusion criteria were (1) patients hospitalised more than 14 days after the stroke for neurological rehabilitation in the neurological rehabilitation department, (2) patients who could not be followed up or whose current medical records could not be obtained to confirm whether there was a recurrent CVD incident over the 5-year follow-up period, and (3) not use of the prescribed pharmacotherapy of chronic diseases by patients.

### 3.2. ICF Profile

The individual categories of the risk factors included in the analysis were assigned relevant code numbers and qualifiers as per the ICF classification.

The effect of depressive disorders on the risk of a recurrent CVD event was assessed using ICF category b152: emotional functions. The following Beck Depression Inventory (BDI) scores were used to measure the severity of depression [26]: qualifier 0: BDI total score 0 to 11—no depression; qualifier 2: BDI total score 12 to 19—mild depression; qualifier 3: BDI total score 20 to 25—moderate depression; qualifier 4: BDI total score 26 to 63—severe depression.

The effect of sleep disturbance on the risk of a recurrent CVD event was assessed using ICF category b134: sleep functions. The following criteria were used to measure the severity of insomnia [7]: qualifier 0—no sleep disturbance (sleep time 6–9 h); qualifier 4—sleep disturbance (sleep time < 6 or > 9 h).

The increased risk of CVD related to heart rate (HR) was estimated using ICF category b4100: heart rate. The following criteria were used to quantify heart rate disorders [8]: qualifier 0—HR < 80/min; qualifier 4—HR >80/min. Heart rhythm disorders were encoded as ICF category b4101: heart rhythm. The following criteria were used [9]: qualifier 0—normal sinus rhythm; qualifier 4—atrial fibrillation.

The effect of carotid artery stenosis on the risk of a recurrent CVD event was assessed using ICF category b4150: functions of arteries. The following criteria were used [10]: qualifier 0—<50% carotid stenosis; qualifier 3—50% to 69% carotid stenosis; qualifier 4—>70% carotid stenosis.

The effect of increased blood pressure (BP) on the risk of a recurrent CVD event was assessed using ICF category b4200: increased blood pressure. The following BP values were used [27]: qualifier 0—BP < 130/80 mm/Hg; qualifier 1—BP > 130/80 mm/Hg; qualifier 2—BP > 140/90 mm/Hg; qualifier 3—BP > 160/90 mm/Hg; qualifier 4—BP > 180/110 mm/Hg.

The effect of liver and renal impairment on the risk of a recurrent CVD event was assessed using ICF category b4301: metabolite-carrying functions of the blood. The following criteria were used to classify renal impairment [28]: qualifier 0—estimated glomerular filtration (eGFR) > 90 mL/min/1.73 m^2^; qualifier 1—eGFR 60–89 mL/min/1.73 m^2^; qualifier 2—eGFR 30–59 mL/min/1.73 m^2^; qualifier 3—eGFR 15–29 mL/min/1.73 m^2^; qualifier 4—eGFR < 15 mL/min/1.73 m^2^, and liver impairment [13]: qualifier 0—bilirubin level < 2x the upper limit of normal (ULN) and ALT (*alanine* transaminase)/AST (aspartate transaminase)/ALP (alkaline phosphatase) < 3x ULN; qualifier 4—bilirubin level > 2x ULN and ALT/AST/ALP > 3x ULN.

Patients receiving anticoagulants have got increased risk of bleeding [14]. This parameter was encoded as ICF category b4302: functions related to the coagulation of blood. If taking VKA (vitamin K antagonist) following values were used: qualifier 0—NO; qualifier 4—YES. If taking NOAC (non-vitamin K antagonist) following values were used: qualifier 0—NO; qualifier 4—YES.

The effect of impaired glycaemic control on the risk of recurrent CVD event was assessed using ICF category b5401: carbohydrate metabolism. The following HbA1c (glycated haemoglobin 1c) values were used [29]: qualifier 0—HbA1c < 7%; qualifier 4—HbA1c > 7%.

The effect of LDL-C (*low*-*density* lipoprotein cholesterol) levels on the risk of a recurrent CVD event was assessed using ICF category b7302, lipid metabolism. The following LDL-C values were used [30]: qualifier 0—LDL-C < 55 mg/dL; qualifier 2—LDL-C 55 mg/dL−70 mg/dL; qualifier 3—LDL-C 71 mg/dL−115 mg/dL; qualifier 4—LDL-C > 116 mg/dL.

Alcohol consumption is an additional risk factor associated with increased risk of a recurrent CVD event. This risk factor was assessed using ICF category e1100, food: alcohol consumption. The following criteria were used [31]: qualifier 0—alcohol intake per day < 10 g; qualifier 4—alcohol intake per day > 10 g.

The increased risk of CVD related to NSAID (nonsteroidal anti-inflammatory drugs) [18] and to smoking [19] was estimated using ICF categories e1101: drugs, and e1109: products or substances for personal consumption, respectively. The following criteria were used: qualifier 0—NO; qualifier 4—YES.

In the following stage, to better highlight any differences which might be present, the graphical summary included a percentage distribution of the qualifiers of risk factor categories as per the ICF classification: qualifier 0, dark green—no risk factors if the value of the percentage distribution was in the range of 0% to 4%; qualifier 1, light green—low risk factor if the value of the percentage distribution was between 5% and 24%; qualifier 2, yellow—moderate risk factor if the value of the percentage distribution was 25–49%; qualifier 3, orange—high risk factor if the value of the percentage distribution was 50–95%; qualifier 4, red—extremely high risk factor if the value of the percentage distribution was 96–100%.

### 3.3. Statistical Analysis

The data analysis was carried out using Statistica v. 13.1. The parameters of descriptive statistics are reported as mean values with standard deviations (SD) and median, minimum, and maximum levels. The categorical variables are presented as counts and frequencies. The Shapiro–Wilk test was used to assess the normality of the distribution of test scores. Non-parametric analyses were used when the data were found not to meet the assumptions defined for parametric analysis. The significance of differences between results or both groups was evaluated based on the parametric Student’s *t*-test for independent variables or the non-parametric Mann–Whitney test. The chi-squared test was used to compare differences between groups in terms of categorical variables. *p*-values less than 0.05 were considered to be statistically significant.

## 4. Results

### 4.1. Characteristics of the Study Groups

The group after IS was consisted of 55 patients, the group after ICH was consisted of 47 patients. The study groups were significantly different in terms of the age of patients at the time of stroke (*p* < 0.002). The average age of post-IS patients was 69.3 years (SD ± 12.5), whilst the average age of post-ICH patients was 61.3 years (SD ± 12.6). The majority of post-IS patients were women (50.90%) whilst the majority of post-ICH patients were men (57.40%). As compared to post-ICH patients, post-IS patients were significantly more frequently observed to have had recurrent CVD incidents (*p* < 0.001), including recurrent IS (*p* = 0.031) and myocardial infarction (*p* < 0.019). Additionally, recurrent CVD incidents were more often fatal (*p* = 0.003) in this patient group. Detailed characteristics of the study groups are shown in Table 1.

### 4.2. Recurrence of CVD Incidents According to Age and Gender

Table 2 presents the results of the analysis of the incidence of recurrent CVD incidents over a 5-year follow-up period according to the clinical type of previous stroke, taking into account age and gender. Fatal CVD incidents were significantly more frequently observed among male patients in the post-IS group than male patients in the post-ICH group (*p* = 0.030). Additionally, post-ICH patients over 65 years of age (*p* = 0.016) and male patients (*p* = 0.015) were more frequently observed to not have any recurrent CVD incidents over a 5-year follow-up period than post-IS patients.

### 4.3. Risk Factors for Recurrent CVD Incidents

Table 3 shows the incidence of recurrent CVD incidents in post-IS or post-ICH patients, taking into account the identified risk factors.

Post-IS patients who suffered recurrent CVD incidents were significantly more frequently observed to have atrial fibrillation (*p* = 0.004), abnormal glycosylated haemoglobin levels (*p* = 0.018), and LDL levels above 116 mg/dL (*p* < 0.001), as well as to more often use NSAIDs (*p* < 0.001) as compared to post-ICH patients, who were significantly more frequently observed to have LDL levels in the range of 55–70 mg/dL (*p* < 0.001).

Post-IS patients who did not suffer a recurrent CVD incident were significantly more frequently observed to have atrial fibrillation (*p* = 0.046) and LDL levels above 116 mg/dL (*p* = 0.008), as well as to more often use NSAIDs (*p* < 0.001) as compared to post-ICH patients, who were more frequently observed to have LDL levels in the range of 55–70 mg/dL (*p* = 0.014).

Post-IS patients who suffered a recurrent CVD incident were significantly more frequently observed to have abnormal arterial blood pressure above 140/90 mmHg than patients who suffered no recurrent CVD incidents (*p* = 0.045).

Post-ICH patients who suffered a recurrent CVD incident were significantly more frequently observed to have carotid artery stenosis in the range of 50–69% (*p* = 0.028) and glomerular filtration rates below 15 mL/min/1.73 m^2^ (*p* < 0.001) as compared to patients who had no recurrent CVD incidents.

#### 4.3.1. Ischemic Stroke

For patients with extremely high risk factors, post-IS patients who suffered a recurrent CVD incident were more frequently observed to have depression, insomnia, abnormal heart rate and rhythm, carotid artery stenosis over 70%, elevated arterial blood pressure (above 180/110 mmHg), glomerular filtration rate below 15 mL/min/1.73 m^2^, abnormal glycosylated haemoglobin levels, and LDL levels higher than 116 mg/dL and to smoke and abuse alcohol, as compared to patients with no recurrent CVD incidents.

For those with a significant risk factor, carotid artery stenosis in the range of 50% to 69%, glomerular filtration rates in the range of 15 to 29 mL/min/1.73 m^2^, and LDL levels in the range of 71 mg/dL to 115 mg/dL were observed more frequently.

In terms of a moderate risk factor, glomerular filtration rates in the range of 30 to 59 mL/min/1.73 m^2^ and elevated arterial blood pressure (above 140/90 mmHg) were more frequently observed.

#### 4.3.2. Haemorrhagic Stroke

For patients with extremely high risk factors, post-ICH patients who suffered a recurrent CVD incident were more frequently observed to have depression, abnormal heart rhythm, carotid artery stenosis over 70%, elevated arterial blood pressure (above 180/110 mmHg), and abnormal glycosylated haemoglobin levels and to smoke and use NOACs, VKAs, and NSAIDs, as compared to patients with no recurrent CVD incidents.

In terms of significant risk factors, carotid artery stenosis ranging from 50% to 69% and LDL levels in the range of 71 to 115 mg/dL were observed more frequently.

For those with moderate risk factors, elevated arterial blood pressure (above 140/80 mmHg), glomerular filtration rate in the range of 30 to 59 mL/min/1.73 m^2^, and LDL levels ranging from 55 to 70 mg/dL were more frequently observed.

#### 4.3.3. Ischemic Stroke vs. Haemorrhagic Stroke

For patients with extremely high risk factors, post-IS patients who suffered a recurrent CVD incident were more frequently observed to have abnormal heart rate and rhythm, carotid artery stenosis over 70%, glomerular filtration rates below 15 mL/min/1.73 m^2^, LDL levels higher than 116 mg/dL, and abnormal glycosylated haemoglobin levels and liver function test results and to smoke and use NSAIDs and NOACs as compared to post-ICH patients. On the other hand, post-ICH patients were significantly more frequently observed to suffer from depression, insomnia, elevated arterial blood pressure (above 180/110 mmHg), and alcohol abuse. The frequency of the use of VKAs was found to be similar in both groups.

For those with significant risk factors, carotid artery stenosis was observed to be in the range of 50% to 69%, in both the post-IS and the post-ICH groups. On the other hand, post-IS patients were more often observed to have glomerular filtration rates in the range of 15 to 29 mL/min/1.73 m^2^ and LDL levels ranging from 71 to 115 mg/dL. Post-IS patients, however, were more frequently observed to have elevated arterial blood pressure (exceeding 160/90 mmHg).

In terms of a moderate risk factor, post-IS patients were observed more often than post-ICH patients to have glomerular filtration rates in the range of 30 to 59 mL/min/1.73 m^2^. On the other hand, post-ICH patients were more frequently observed to have elevated arterial blood pressure (above 140/80 mmHg) and LDL levels in the range of 55 to 70 mg/dL.

### 4.4. CVD Risk Factor Profile in Secondary Prevention according to the ICF Classification

Table 4 shows a list of categories of the ICF classification and the percentage distribution of risk factors for CVD in secondary prevention according to the recurrence of CVD incidents and the clinical type of stroke.

### 4.5. Coexistence of CVD Risk Factors

Table 5 shows a comparison of the cumulative risk of CVD in the study groups. In both post-IS patients (*p* = 0.031) and post-ICH patients (*p* = 0.002) who had a recurrent CVD incident, significantly more risk factors for CVD were identified than for patients who had no recurrent CVD incidents.

**Figure 2:**
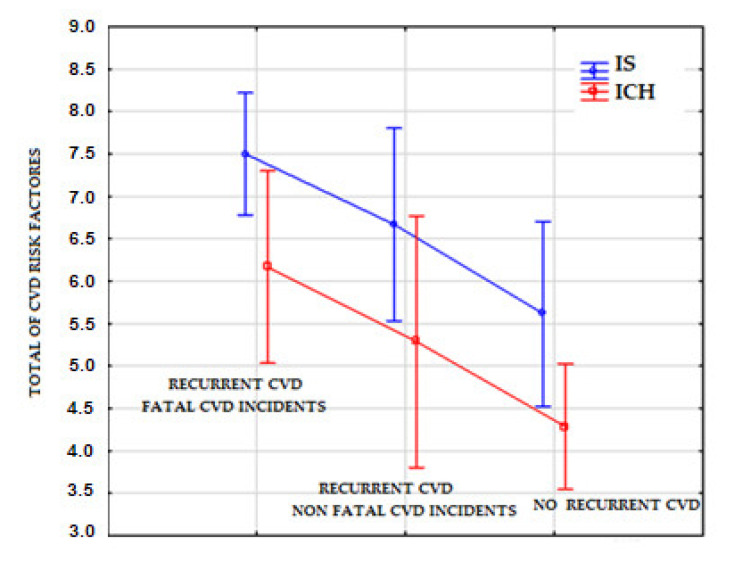
Distribution of the total of CVD risk factors. CVD—cardiovascular disease; ICF—International Classification of Functioning, Disability and Health; IS—ischemic stroke.

The values of distribution of the total risk factors for CVD were observed to be higher for the post-IS group than for the post-ICH group (Figure 2). In both groups, the greatest values of distribution of risk factors for CVD were observed for patients following a fatal recurrent CVD incident, whilst the lowest values were for patients who had no recurrent CVD incidents.

## 5. Discussion

Sequelae of cardiovascular disease are a major cause of death around the world and more than half of the patients with a history of stroke are at an increased risk of recurrent CVD incidents, including recurrent stroke in particular [1]. In our study (Table 1), as many as 76% of post-IS patients and only 40% of post-ICH patients (*p* < 0.001) had recurrent CVD incidents over the 5-year follow-up period. Our results are consistent with those reported by Vickrey et al. [3], where the risk of recurrent stroke over a 5-year follow-up period was greater than 40%. According to Yamamoto et al. [2], recurrent stroke incidents in post-IS patients most often have the same etiopathogenesis and the risk of future myocardial infarction is 15%. Only 5% of all incidents are classified as IS. On the other hand, 42% of post-ICH patients are observed to have recurrent IS. In the present study (see Table 1), recurrent IS episodes were observed in more than 60% of patients with a history of primary IS and in 40% of patients with a history of primary ICH (*p* = 0.031). No recurrent IS was observed in either group. Additionally, more than 14% of the post-IS patients had myocardial infarction, whilst no cases of myocardial infarction were identified in the post-ICH group (*p* < 0.019). Yamamoto et al. [2] demonstrated that more than half of the patients who suffered a recurrent stroke are at risk of death [2]. This is consistent with our results, where recurrent CVD incidents in the post-IS group were fatal in more than 54% of patients. In this study, a significantly lower mortality rate (*p* = 0.003) and a lower incidence of recurrent CVD incidents (*p* < 0.001) were observed in patients with a history of primary ICH. In our opinion, these differences are due to the pathogenesis and the more severe clinical presentation of IS as compared to ICH [32]. Moreover, there was no difference in the occurrence of a recurrent CVD incident and the mortality due to a recurrent CVD incident in a 5-year follow-up depending on the conservative or surgical treatment in both IS and ICH. The obtained results are consistent with the studies by McCarthy et al. [33], who proved that treatment with mechanical thrombectomy in IS does not reduce long-term mortality compared to conservative treatment. Moreover, Hemphill et al. [34] proved that surgical treatment in patients after ICH did not show any clear benefits compared to conservative treatment. In terms of age and sex (Table 2), fatal recurrent CVD events were significantly more frequently observed in men with a history of primary IS (*p* = 0.030). As proved by Zhang et al. [32], the incidence of IS in men aged over 55 was more than two times higher than in the case of ICH, which may result in higher mortality in men after IS in this age group compared to ICH. The absence of recurrent cardiovascular events was significantly more often observed after ICH in men (*p* = 0.015) and over 65 (*p* = 0.016). The research results correspond to the results obtained by Zhang et al. [35].

As argued by Adams et al., the more risk factors that are identified, the greater the likelihood of a recurrent CVD incident [21]. As compared to patients with no recurrent CVD incidents, significantly more coexisting risk factors for CVD were observed both in post-IS patients (*p* = 0.031) and post-ICH patients (*p* = 0.002) who had a recurrent CVD incident (Table 5). Additionally, in all three options included in the analysis, higher values of distribution of the total risk factors for CVD were observed for post-IS patients than for post-ICH patients (Figure 2). This is due to the fact that post-IS patients have more coexisting diseases [36]. Therefore, for the purposes of secondary prevention, it is reasonable to monitor patients for various risk factors simultaneously. In our study (Table 3), post-IS patients who had a recurrent CVD incident were significantly more frequently observed to have atrial fibrillation than the post-ICH patients (*p* < 0.004). This is consistent with the results obtained by Lip et al. [9], who determined that post-IS atrial fibrillation was associated with a high risk of recurrent CVD incidents. Another risk factor included in the analysis was abnormal glycosylated haemoglobin level. As evidenced by Wu et al., it is significantly more frequently associated with the recurrence of CVD incidents in post-IS patients than in post-ICH patients [15]. Our findings were similar (*p* = 0.018). Post-IS patients who suffered a recurrent CVD incident were also significantly more frequently observed to have abnormal LDL levels (above 116 mg/dL; *p* < 0.001), and were more rarely observed to have LDL levels in the range of 55–70 mg/dL than post-ICH patients (*p* < 0.001). High LDL levels are a strong predictor of the recurrence of CVD incidents. The relationship between dyslipidaemia and the recurrence of CVD incidents due to atherosclerosis is well evidenced [37]. Patients in the post-IS group were also observed to more frequently use NSAIDs (*p* < 0.001), which are associated with a higher risk of intracerebral haemorrhage [18]. In the post-IS group, patients who suffered a recurrent CVD incident were significantly more frequently observed to have elevated arterial blood pressure (*p* = 0.045). Lower arterial blood pressure in post-stroke patients reduces the risk of recurrent CVD incidents [11]. Post-ICH patients who suffered a recurrent CVD incident were significantly more frequently observed to have carotid artery stenosis in the range of 50–69% (*p* = 0.028) and glomerular filtration rates below 15 mL/min/1.73 m^2^ (*p* < 0.001). Significant carotid artery stenosis and an abnormal glomerular filtration rate both increase the risk of recurrent CVD incidents [10,12].

When using the ICF classification in secondary prevention of recurrent CVD incidents, specific attention should be paid to the risk factors associated with extremely high incidence (qualifier 4), which are marked in red (Table 4). Common factors in post-IS and post-ICH patients which are of particular relevance to the recurrence of CVD incidents include depression, abnormal heart rate and rhythm, carotid artery stenosis above 70%, arterial blood pressure higher than 180/110 mmHg, abnormal glycosylated haemoglobin levels, and smoking. Additional risk factors in post-IS patients include insomnia, a glomerular filtration rate below 15 mL/min/1.73 m^2^, LDL levels higher than 116 mg/dL, and alcohol abuse. In post-ICH patients, the use of NOACs, VKAs, and NSAIDs is an additional risk factor of relevance to the recurrence of CVD incidents.

The percentage distribution of risk factors for recurrent CVD incidents according to the categories of the ICF classification, as shown in Table 4, provides information on risk factors and their incidence based on the type of previous stroke. The use of a single tool for monitoring various risk factors for CVD—in the form of the ICF questionnaire—could help to increase the effectiveness of secondary prevention and thus to reduce the risk of recurrent CVD incidents. The simultaneous presentation of several categories of risk factors in the form of ‘dynamic graphs’ makes it possible to analyse them in a legible and concise manner, which can help in taking appropriate clinical decisions.

## 6. Limitations

The limitations of our study were retrospective character of the study and relatively small number of study groups. Additionally, as concerns the analysis of individual risk factors, the study groups were composed of different numbers of patients. We have not also included BMI as a known risk factor for recurrent CVD incidents due to the lack of data in the available medical records from which BMIs could be calculated.

## 7. Conclusions

The type of primary stroke determines the type and frequency of risk factors and the frequency of recurrence of CVD events over the 5-year follow-up period. Patients after IS have a higher risk of recurrence of CVD events, including fatal ones in the 5-year follow-up compared to patients after ICH. In addition, post-IS patients who have a recurrent CVD event over a 5-year follow-up have more risk factors for a CVD event than ICH. ICF can be useful in assessing and analysing risk factors for recurrent CVD events, which can help improve the effectiveness of secondary prevention.

## Figures and Tables

**Table 1:** Characteristics of the study groups.

Parameter	Variable	IS	ICH	*p*
Gender	Female *n* (%)	28 (50.9%)	20 (42.6%)	0.399 ^a^
	Male *n* (%)	27 (49.1%)	27 (57.4%)	
Age	Mean ± SD (years)	69.3 ± 12.5	61.3 ± 12.6	0.002 ^b^
	Median (years)	71.6	63.0	
	Min–Max (years)	43.0–93.0	27.0–81.0	
Treatment of stroke	Conservative *n* (%)	46 (83.6%)	36 (76.6%)	0.375 ^a^
	Interventional *n* (%)	9 (16.4%)	11 (23.4%)	
Conservative *n* (%)	Fatal incident CVD	26 (86.7%)	12 (100.0%)	0.184 ^a^
	Recurrent CVD incident	10 (83.3%)	4 (57.1%)	0.211 ^a^
	No recurrent CVD incident	10 (76.9%)	20 (71.4%)	0.712 ^a^
Interventional *n* (%)	Fatal incident CVD	4 (13.3%)	0 (0.0%)	0.184 ^a^
	Recurrent CVD incident	2 (16.7%)	3 (42.9%)	0.211 ^a^
	No recurrent CVD incident	3 (23.1%)	8 (28.6%)	0.712 ^a^
Time from first stroke to recurrent CVD incident	Mean ± SD (months)	36.6 ± 24.9	36.6 ± 24.5	0.963 ^c^
	Median (months)	36.0	36.0	
	Min–Max (months)	4.0–84.0	12.0–108.0	
Recurrent CVD incident:	*n* (%)	42 (76.4%)	19 (40.4%)	<0.001 ^a^
IS	*n* (%)	34 (61.8%)	19 (40.4%)	0.031 ^a^
Myocardial infarction	*n* (%)	8 (14.5%)	0 (0.0%)	<0.019 ^a^
Fatal incident	*n* (%)	30 (54.5%)	12 (25.5%)	0.003 ^a^
No recurrent CVD incident	*n* (%)	13 (23.6%)	28 (59.6%)	<0.001 ^a^

CVD—cardiovascular disease; *n*: size of sample; IS—ischemic stroke; ICH—haemorrhagic stroke; SD—standard deviation; ^a^ chi-squared test; ^b^ Student’s *t*-test for independent variables; ^c^ Mann–Whitney test.

**Table 2:** Analysis of the recurrence of CVD incidents, according to age and gender.

Recurrent CVD Incident	YES						NO		
	Fatal CVD Incidents			Non Fatal CVD Incidents					
Patients Groups	IS	ICH	*p*	IS	ICH	*p*	IS	ICH	*p*
<65 years	8 (38.1%)	5 (17.9%)	0.206 ^a^	6 (28.6%)	6 (21.4%)	0.730 ^a^	7 (33.3%)	17 (60.7%)	0.102 ^a^
≥65 years	22 (64.7%)	7 (36.8%)	0.099 ^a^	6 (17.7%)	1 (5.3%)	0.440 ^a^	6 (17.7%)	11 (57.9%)	0.016 ^a^
Female	34 (46.4%)	5 (25.0%)	0.267 ^a^	7 (25.0%)	3 (15.0%)	0.608 ^a^	8 (28.6%)	12 (60.0%)	0.061 ^a^
Male	17 (63.0%)	7 (26.0%)	0.030 ^a^	5 (18.5%)	4 (14.8%)	0.836 ^a^	5 (18.5%)	16 (59.3%)	0.015 ^a^

CVD—cardiovascular disease; F—female, M—male; IS—ischemic stroke; ICH—haemorrhagic stroke; ^a^ chi-squared test.

**Table 3:** Analysis of the incidence of risk factors for CVD in secondary prevention, according to the clinical type of the previous stroke.

ICF Category	Patients Groups	IS	ICH	*p*	IS	ICH	*p*	IS		*p*	ICH		*p*
	Recurrent CVD Incident	YES *n* = 42	YES *n* = 19		NO *n* = 13	NO *n* = 28		YES *n* = 42	NO *n* = 13		YES *n* = 19	NO *n* = 28	
b 152 Emontional functions—Depression	*n* (%)	10 (23.8%)	6 (31.6%)	0.521	2 (15.4%)	7 (25.0%)	0.490	10 (23.8%)	2 (15.4%)	0.522	6 (31.6%)	7 (25.0%)	0.620
b 134 Sleep functions Insomia	*n* (%)	10 (23.8%)	7 (36.8%)	0.294	2 (15.4%)	11 (39.3%)	0.126	10 (23.8%)	2 (15.4%)	0.522	7 (36.8%)	11 (39.3%)	0.863
b 4100 Heart rate	HR > 80/min, *n* (%)	20 (48.8%)	7 (36.8%)	0.383	5 (38.5%)	11 (39.3%)	0.961	20 (48.8%)	5 (38.5%)	0.515	7 (36.8%)	11 (39.3%)	0.863
b 4101 Heart rhythm	Atrial fibrillation, *n* (%)	23 (54.8%)	3 (15.8%)	0.004	4 (30.8%)	2 (7.1%)	0.046	23 (54.8%)	4 (30.8%)	0.130	3 (15.8%)	2 (7.1%)	0.342
b 4150 Functions of arteries	Stenosis 50–69%, *n* (%)	12 (28.6%)	6 (31.6%)	0.812	3 (23.1%)	2 (7.1%)	0.145	12 (28.6%)	3 (23.1%)	0.697	6 (31.6%)	2 (7.1%)	0.028
	Stenosis > 70%, *n* (%)	14 (33.3%)	2 (10.5%)	0.061	1 (7.7%)	0 (0.0%)	0.137	14 (33.3%)	1 (7.7%)	0.070	2 (10.5%)	0 (0.0%)	0.080
b 4200 Increased blood pressure	>140/90 mmHg, *n* (%)	40 (95.2%)	18 (94.7%)	0.937	10 (76.9%)	23 (82.1%)	0.696	40 (95.2%)	10 (76.9%)	0.045	18 (94.7%)	23 (82.1%)	0.205
b 4302 Metabolite-carrying functions of the blood	eGFR(mL/min/1.73 m^2^) <15, *n* (%)	6 (14.3%)	6 (31.6%)	0.116	0 (0.0%)	0 (0.0%)	1.0	6 (14.3%)	0 (0.0%)	0.149	6 (31.6%)	0 (0.0%)	0.001
	Bilirubin >2x ULN, ALT/AST/ALP > 3x ULN, *n* (%)	4 (9.5%)	1 (5.3%)	0.580	1 (7.7%)	4 (14.3%)	0.548	4 (9.5%)	1 (7.7%)	0.844	1 (5.3%)	4 (14.3%)	0.327
b 4303 Clotting functions.	VKA, *n* (%)	8, (19.0%)	3 (15.8%)	0.763	3 (23.1%)	2 (7.1%)	0.145	8 (19..0%)	3 (23.1%)	0.747	3 (15.8%)	2 (7.1%)	0.342
	NOAC, *n* (%)	7 (16.7%)	1 (5.3%)	0.222	2 (15.4%)	1 (3.6%)	0.178	7 (16.7%)	2 (15.4%)	0.912	1 (5.3%)	1 (3.6%)	0.778
b 5401 Carbohydrate metabolism	HbA1 > 7%, *n* (%)	20 (47.6%)	3 (15.8%)	0.018	3 (23.1%)	4 (14.3%)	0.486	20 (47.6%)	3 (23.1%)	0.118	3 (15.8%)	4 (14.3%)	0.887
b 7302 Lipid metabolism	LDL 55–70 mg/dL, *n*(%)	3 (7.1%)	13 (68.4%)	<0.001	3 (23.1%)	18 (64.3%)	0.014	3 (7.1%)	3 (23.1%)	0.106	13 (68.4%)	18 (64.3%)	0.771
	LDL 71–115 mg/dL, *n* (%)	17 (40.5%)	5 (26.3%)	0.285	3 (23.1%)	2 (7.1%)	0.145	17 (40.5%)	3 (23.1%)	0.255	5 (26.3%)	2 (7.1%)	0.069
	LDL > 116 mg/dL, *n*(%)	22 (52.4%)	0 (0.0%)	<0.001	3 (23.1%)	0 (0.0%)	0.008	22 (52.4%)	3 (23.1%)	0.064	0 (0.0%)	0 (0.0%)	1.0
e1100 Food	Alcohol consumption > 10 g (>1 unit), *n* (%)	01 (2.4%)	1 (5.3%)	1.0	0 (0.0%)	2 (7.1%)	0.325	1 (2.4%)	0 (0.0%)	0.573	1 (5.3%)	2 (7.1%)	0.804
e 1101 Drugs	NSAIDs, *n* (%)	34 (81.0%)	3 (15.8%)	<0.001	12 (92.3%)	4 (14.3%)	<0.001	34 (81.0%)	12 (92.3%)	0.335	3 (15.8%)	4 (14.3%)	0.887
e1109 Products or substances for personal consumption, other specified	Smoking, *n* (%)	16 (38.1%)	6 (31.6%)	0.671	3 (23.1%)	5 (17.9%)	0.696	16 (38.1%)	3 (23.1%)	0.320	6 (31.6%)	5 (17.9%)	0.277

ALT—alanine aminotransferase; AST—aspartate aminotransferase; ALP—alkaline phosphatase; BP—blood pressure; CVD—cardiovascular disease; eGFR—estimated glomerular filtration rate; ICH—intracerebral haemorrhage; ICF—International Classification of Functioning, Disability and Health; IS—ischemic stroke; HbA1c—glycated haemoglobin 1c; HR—heart rate; LDL-C—low-density lipoprotein cholesterol; n—size of the sample; NOAC—nonvitamin K antagonist oral anticoagulants; NSAIDs—nonsteroidal anti-inflammatory drugs; ULN—upper limit of normal; VKA—vitamin K antagonist.

**Table 4:**
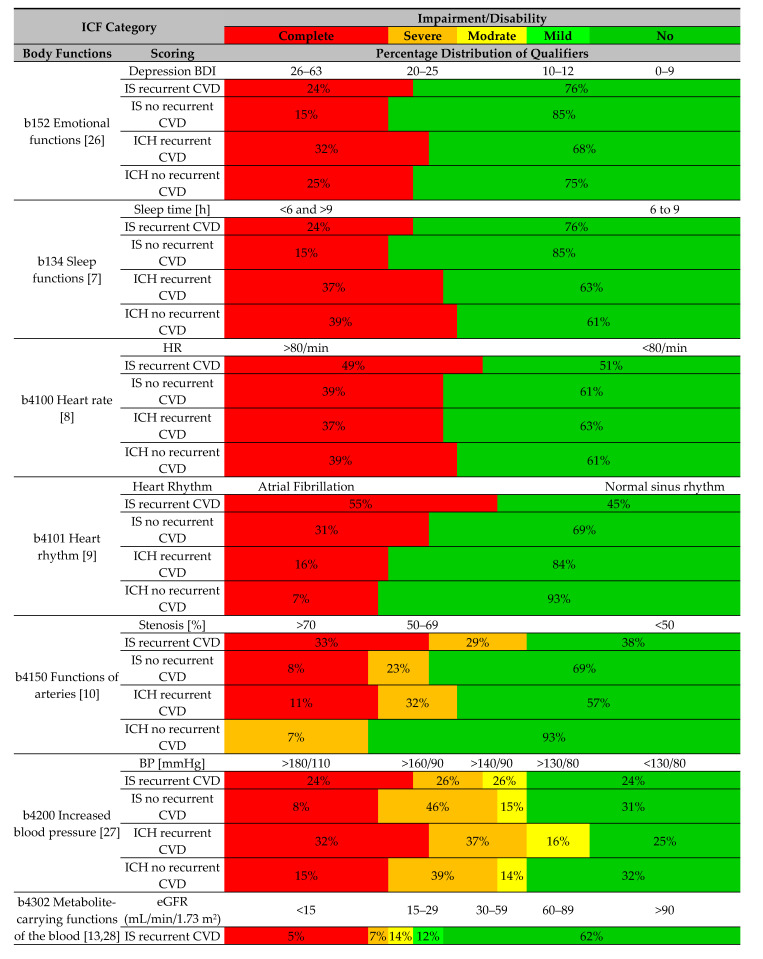

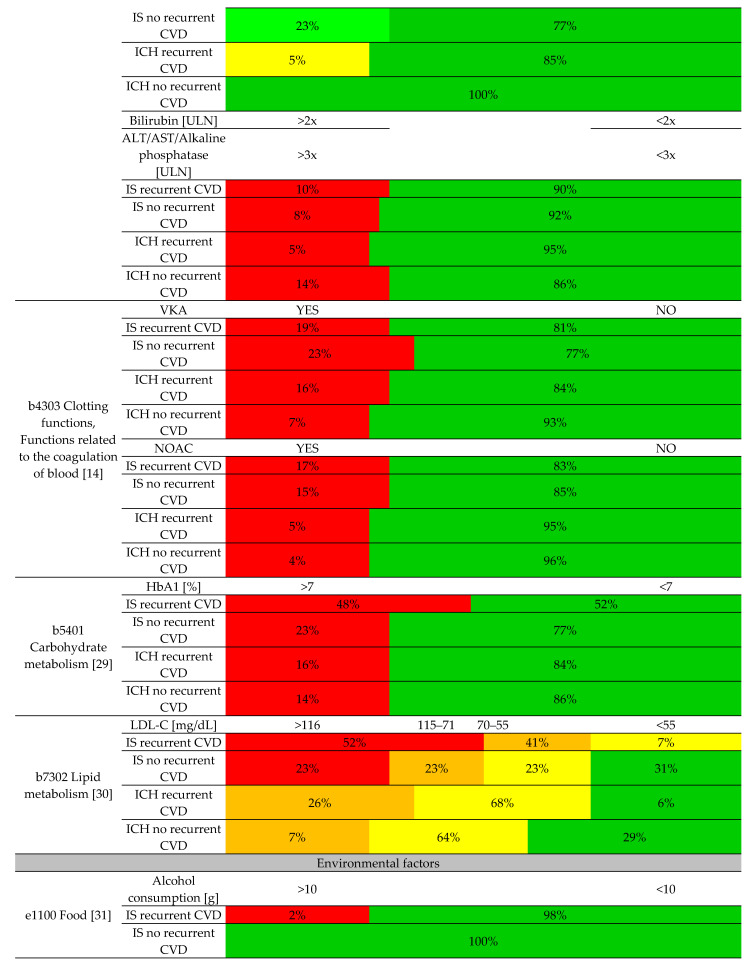

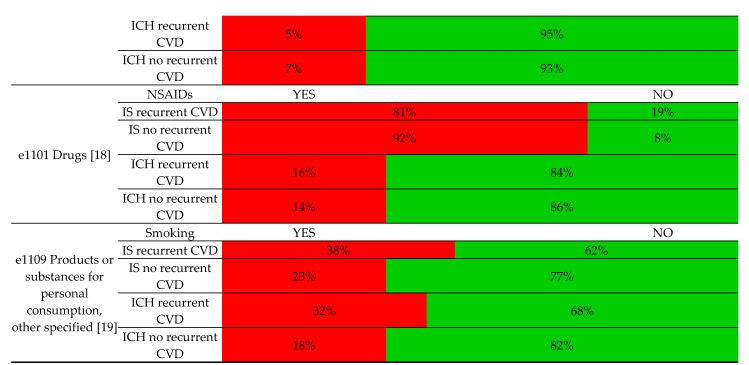
Profile of CVD risk factors as per categories of the ICF classification.

ALT—*alanine* aminotransferase; AST—aspartate aminotransferase; ALP—alkaline phosphatase; BP—blood pressure; CVD—cardiovascular disease; eGFR—estimated *glomerular filtration rate*; ICH—intracerebral *haemorrhage*; ICF—International Classification of Functioning, Disability and Health; IS—ischemic stroke; HbA1c—glycated haemoglobin 1c; HR—heart rate; LDL-C—*low*-*density* lipoprotein cholesterol; n—size of the sample; NOAC—nonvitamin K antagonist oral anticoagulants; NSAIDs—nonsteroidal anti-inflammatory drugs; ULN—upper limit of normal; VKA—vitamin K antagonist; Red color—extreme problems; Orange color—significant problems; Yellow color—moderate problems; Light green color—minor problems; Dark green color—no problems.

**Table 5:** Comparison of the total incidence of CVD risk factors.

Risk Factors for Recurrent CVD Incident						
Patients Groups	IS		*p*	ICH		*p*
Recurrent CVD Incident	YES	NO		YES	NO	
Mean ± SD	7.3 ± 2.0 *	5.6 ± 3.0	0.031 ^a^	5.8 ± 1.6	4.3 ± 1.6 *	0.002 ^b^
Median	7.0	4.0		6.0	4.0	
Min–Max	4.0–12.0	2.0–11.0		3.0–8.0	1.0–7.0	

CVD—cardiovascular disease; IS—ischemic stroke; ICH—haemorrhagic stroke; SD—standard deviation; ^a^ Mann–Whitney test; ^b^ Student’s *t*-test for independent variables; *—Wilcoxon test.

## Data Availability

The data presented in this study are available on request from the first author. The data are not publicly available due to ethical restrictions.
